# A novel and independent method for time‐resolved gantry angle quality assurance for VMAT

**DOI:** 10.1002/acm2.12129

**Published:** 2017-07-13

**Authors:** Todsaporn Fuangrod, Peter B. Greer, Benjamin J. Zwan, Michael P. Barnes, Joerg Lehmann

**Affiliations:** ^1^ Department of Radiation Oncology Calvary Mater Newcastle Hospital Newcastle NSW Australia; ^2^ School of Mathematical and Physics Sciences Faculty of Science and IT the University of Newcastle Newcastle NSW Australia; ^3^ Central Coast Cancer Centre Gosford Hospital Gosford NSW Australia; ^4^ School of Medical Radiation Sciences University of Newcastle Newcastle NSW Australia

**Keywords:** gantry angle, quality assurance, VMAT

## Abstract

Volumetric‐modulated arc therapy (VMAT) treatment delivery requires three key dynamic components; gantry rotation, dose rate modulation, and multi‐leaf collimator motion, which are all simultaneously varied during the delivery. Misalignment of the gantry angle can potentially affect clinical outcome due to the steep dose gradients and complex MLC shapes involved. It is essential to develop independent gantry angle quality assurance (QA) appropriate to VMAT that can be performed simultaneously with other key VMAT QA testing. In this work, a simple and inexpensive fully independent gantry angle measurement methodology was developed that allows quantitation of the gantry angle accuracy as a function of time. This method is based on the analysis of video footage of a “Double dot” pattern attached to the front cover of the linear accelerator that consists of red and green circles printed on A4 paper sheet. A standard mobile phone is placed on the couch to record the video footage during gantry rotation. The video file is subsequently analyzed and used to determine the gantry angle from each video frame using the relative position of the two dots. There were two types of validation tests performed including the static mode with manual gantry angle rotation and dynamic mode with three complex test plans. The accuracy was 0.26° ± 0.04° and 0.46° ± 0.31° (mean ± 1 SD) for the static and dynamic modes, respectively. This method is user friendly, cost effective, easy to setup, has high temporal resolution, and can be combined with existing time‐resolved method for QA of MLC and dose rate to form a comprehensive set of procedures for time‐resolved QA of VMAT delivery system.

## 1 INTRODUCTION

Volumetric‐modulated arc therapy (VMAT) is a modern radiation treatment technique that allows a precise three‐dimensional (3D) radiation dose to be delivered as the gantry rotates through one or more arcs.^1^, ^2^, ^3^ Compared to intensity‐modulated radiation therapy (IMRT), where the gantry is static during dose delivery, VMAT offers more precise target coverage with lower monitor units (MU).^3^, ^4^ Furthermore, the delivery times for VMAT treatments are shorter compared to IMRT treatments,^5^ potentially reducing the intra‐fractional patient movement during deliveries.^6^ VMAT deliveries require a complex treatment plan, involving three key dynamic components; (a) gantry rotation, (b) dose rate modulation, and (c) multi‐leaf collimator (MLC) motion, which are all simultaneously varied during the delivery.^2^, ^3^, ^4^, ^5^, ^6^, ^7^

It is essential to develop an independent quality assurance (QA) program for these three components of VMAT deliveries.^8^ Commissioning and QA procedures for VMAT can be conducted using electronic portal imaging device (EPID). One of the most commonly used QA procedures for Varian linacs (Varian Medical Systems, Palo Alto, CA, USA) are the Ling tests, which rely solely on integrated EPID images.^9^ These tests, like most common QA methodologies, focus on the accuracy of the dose rate and MLC control systems rather than the gantry angle and gantry speed aspects of the VMAT delivery. A limitation of these tests is that the EPID panel rotates with the gantry and so the accuracy of the gantry angle during VMAT is not independently assessed. Furthermore, it has been shown that a slight misalignment of the gantry angle could severely affect the dose distribution of VMAT plan deliveries, which could result in serious clinical consequences due to the steep dose gradients and complex MLC shapes involved.^10^, ^11^ For this reason, it is essential that the accuracy of the gantry angle during dynamic arc deliveries is assessed on a regular basis.

Systems for independent gantry QA have been developed by a number of groups. Adamson et al. (2012) proposed the use of a custom‐built phantom with five gold coils of 0.8 cm diameter embedded in Styrofoam. Gantry angle determination was performed by acquiring cine‐EPID images and extracting the projection of the gold coils to determine the gantry angle. The accuracy of this technique was characterized to be 0.0° ± 0.3° for static and 0.2° ± 0.2° for dynamic gantry rotation.^12^ A similar approach was used by Shandiz et al. (2015) utilizing a low‐cost CCD camera attached to the gantry and a 3D phantom on the couch. This method allows the determination of both the source to surface distance (SSD) and gantry angle independently. The precision of gantry angle determination of this method was reported to be 0.43° (mean).^13^ McCowan et al. 2014^14^ developed the radiographic gantry‐phantom and a correction method to boost the accuracy of EPID image read‐out for gantry angle. Here, a boxcar time delay correction method was applied to the gantry angle from cine EPID image headers resulting in an accuracy of 0.10° ± 0.3° (mean ± 1 SD).^14^ These methods are not suitable for regular QA as they are too resource and time‐intensive to be applied on a regular basis. Furthermore, most methodologies rely on the purchase or construction of a custom‐built phantom. A more time‐efficient and easily assessable technique is required in order for radiotherapy centers to perform independent time‐resolved gantry angle QA routinely.

A fast and accurate method for dynamic gantry angle measurements was developed by Rowshanfarzad et al. 2012^11^ who used a ball bearing (BB) phantom placed on the couch and cine‐EPID imaging. The projection of the BB phantom onto EPID images was used to calculate the gantry angle as a function of time. Based on this investigation, the accuracy of gantry angle determination was 0.20° ± 0.16° and 0.05° ± 0.10° (mean ± 1 SD) for static and dynamic gantry rotation using BB phantom, respectively.^11^ This method has potential for use as a routine QA tool with easy setup and low cost of equipment. A limitation of this technique is that it cannot be performed simultaneously with QA of other key VMAT components (i.e., dose rate and MLC) due to the presence of the BB phantom.

Time‐resolved commissioning and QA of VMAT delivery systems have been demonstrated by a number of groups.^15^, ^16^, ^17^, ^18^ Many of these QA procedures, for example, those which rely on machine log files or information within the EPID image header, rely heavily on the gantry angle readout from the linear accelerator itself to synchronize measurements to the treatment plan. This is also the case for a number of EPID‐based patient‐specific QA techniques^19^, ^20^ and delivery verification systems.^21^, ^22^ Although, as the hardware is developing, the EPID image header of TrueBeam linac has been shown to be more accurate,^23^, ^24^ this alone cannot be used for gantry angle QA as it is not independent of the linac control system. The log file and EPID header gantry angle might be accurate while all is working well, but if there is a drift or fault which results in loss of gantry calibration accuracy then this will not be evident in the log file or EPID header. Therefore, it is fully independent the method presented in this study will be sensitive to such a change and hence is suitable for routine QA.

In order for these systems to be fully independent and sensitive to all types of delivery errors (including gantry angle errors), a method is required which can accurately and easily measure the dynamic gantry angle as a function of time during acquisition of EPID images or phantom measurements. In this work, we developed a simple, inexpensive, and fully independent gantry angle measurement methodology that allows quantitation of the gantry angle accuracy as a function of time. Our proposed method is inherently independent of the linear accelerator system and does not impede phantom or EPID‐based QA of MLC and dose rate, which can be performed simultaneously with this measurement. The accuracy of this novel method was evaluated for both static and dynamic gantry angle plans and for test plans designed specifically for dynamic gantry angle QA. The procedures and techniques developed in the work can be used to complement the existing time‐resolved MLC and dose rate QA methods for VMAT control systems.

## 2 METHODS

### 2.A Overview and setup

**Figure 1:**
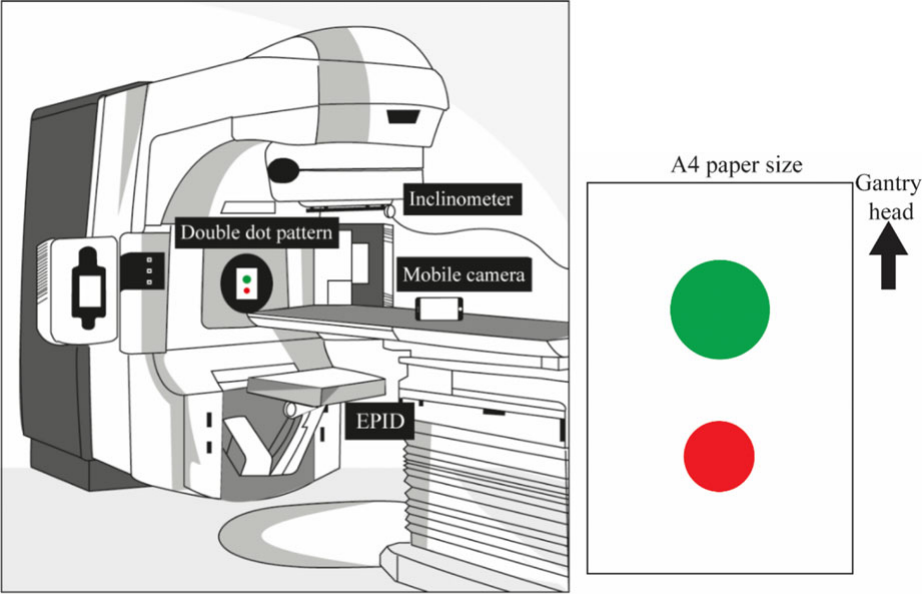
Experimental setup for dynamic gantry angle QA (left) and printed Double Dot pattern (right).

The gantry angle measurement technique is based on the analysis of video footage of a “Double Dot” pattern attached to the front cover of the linear accelerator. The pattern consists of red and green circles of different size printed on an A4 sheet of paper using a conventional printer. Placement on the linear accelerator should be approximately near the axis of rotation but exact position is not critical. A standard mobile phone camera is placed on the couch to record the video footage during gantry rotation. The video file is subsequently analyzed and used to determine the gantry angle from each video frame using the relative position of the two dots (see Fig. 1).

#### 2.B Gantry angle determination

**Figure 2:**
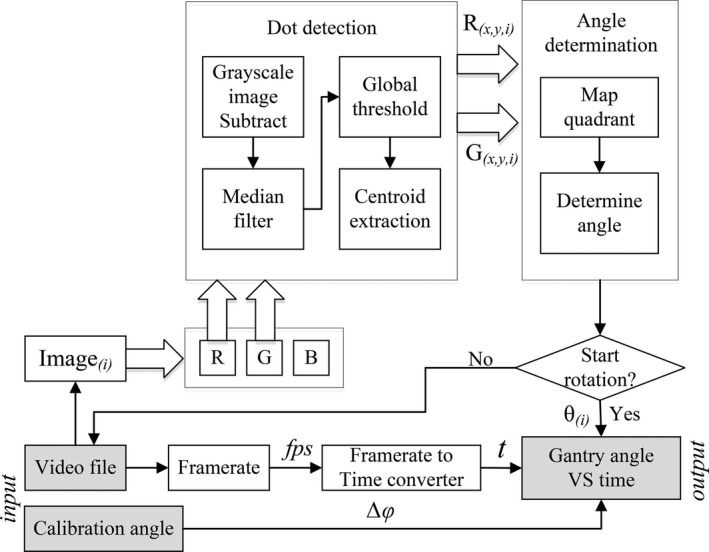
Overview of methodology for gantry angle determination from each image frame of the Double Dot pattern.

Determination of the gantry angle is based on measurement of the relative angle between the red and green dots (see Fig. 1). The method used to extract this angle from each frame of the acquired video footage can be divided into three parts: dot detection, angle determination and, gantry angle calibration. An overview of the algorithm is given in Fig 2, which is discussed in greater detail in the following sections.

#### 2.B.1 Dot detection algorithm

Following acquisition of the video footage, the colourmap matrix (R‐red, G‐green, B‐blue) is extracted from each frame. Image frames corresponding to the red and green channels are used to localize the two dots separately. This effectively filters out other features of the image to maximize the accurate detection of each of the dots. For each image (red and green), the image noise is reduced by applying a median filter with 5 × 5 pixels^2^ window size. Second, a 50% global threshold is used to identify the outline of the circle, which is subsequently used to locate the centroid of the circle.

#### 2.B.2 Angle determination

**Figure 3:**
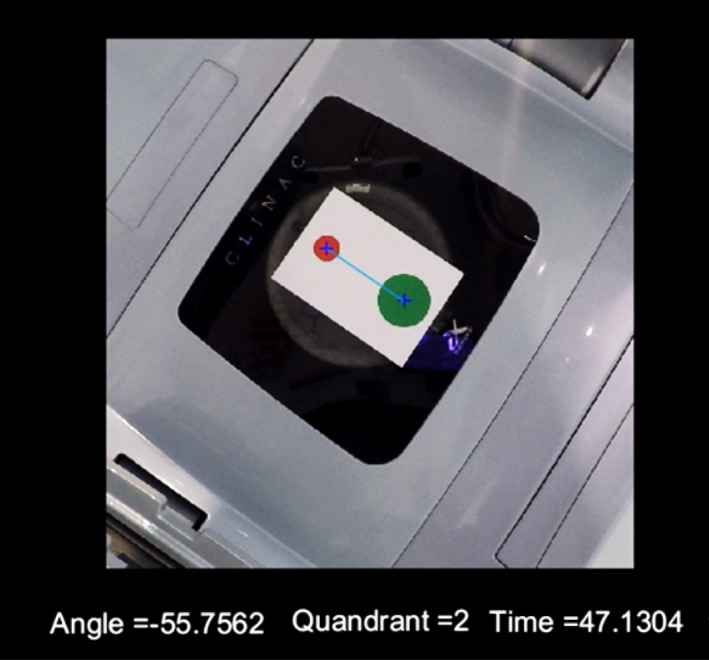
An example of the output of the gantry angle QA system for a single frame of the video footage.

This process outlined above is repeated for both the red and green images. The relative locations of the centroids are first used to determine which of the four quadrants the gantry is in, to differentiate between the actual gantry angle *θ* and the angle *θ* + 180°. Second, the angle between the centroid of the red and green circles is calculated using eq. (1) where R_x_ is the x‐coordinate of the red circle, R_y_ is the y‐coordinate of the red circle, G_x_ is the x‐coordinate of the green circle and, G_y_ is the y‐coordinate of the green circle. An example of the output of the system for a single frame is given in Fig. 3.

(1) $$angle=tan^{-1}\left(\frac{R_{x}-G_{x}}{R_{y}-G_{y}}\right)\cdot \left(\frac{180}{\pi }\right)$$

#### 2.B.3 Gantry angle calibration

After extracting the relative angle between the red and green circles, this angle is then converted into the actual gantry angle. To do this, image frames from the first 10 s of the video footage are used to calculate the calibration angle (*θ*_0_), which is the angle between the two circles when the gantry is at absolute zero. This technique enables the method to be independent of the absolute positioning of the A4 paper on the gantry. The gantry is initially set to absolute 0° using a spirit level rather than relying on the readout of the linear accelerator gantry angle.

### 2.C Delivery system and equipment

All measurements were performed using a Varian 2100 iX linear accelerator (Varian Medical Systems, Palo Alto, CA, USA). The linac control systems relies two encoding potentiometers (one primary and one secondary) that replicate each other and provide signals linearly proportional to the gantry angle.

To test the accuracy of the gantry angle measurement method, three independent methods for gantry angle determination were used: (a) gantry angle from the linac control system recorded within machine log files (DynaLog files), (b) gantry angles from the on‐board imager (OBI) gantry angle encoder, and (c) and independent digital inclinometer.

The DynaLog files are generated by the Varian MLC control software with updated information every 50 ms, from which the gantry angle can be extracted as a function of time. The OBI gantry angle encoder signal is very precise (±0.05°)^19^ and is primarily used for cone‐beam CT image reconstructions. This raw signal was extracted from the header of “dark field” image frames from the KV imager using an existing external frame grabber computer.^17^, ^21^, ^22^ This signal was converted to gantry angle versus time using the known KV image frame rate. As a third independent check of gantry angle, a digital inclinometer, Nordic Transducer NG360 (Hadsund, Denmark) was firmly bolted to a steel frame, which was attached to the gantry head via the accessory tray slot. NG360 is a liquid capacitive‐based inclinometer that contains 0.01° resolution with a range of 360° and maximum readout frequency of 1 Hz. The accuracy has been investigated to be 0.15° ± 0.13° and 1.50° ± 0.22° for static and dynamic gantry rotation tests.^11^ Prior to use, the inclinometer was calibrated at 0° (IEC scale) using a level to compensate for the physical setup error. The device was connected to a PC through a converter and signal was read through the vendor supplied software. It is important to note that such commercial inclinometers are known to have 1° lag for dynamic‐arc deliveries particularly when the gantry is accelerating or decelerating.^11^

The mobile camera used in this experiment to collect the video footage of the Double Dot pattern was a standard Samsung Galaxy S6 mobile phone (Samsung Electronics, South Korea). This device has a 16 megapixel rear camera and contains optical image stabilization and autofocus hardware features. The dimensions of the video are 3840 × 2160 pixels and the frame rate is 30 frames per second.^25^

### 2.D Validation of methodology for static gantry

To assess the accuracy of the Double Dot method for gantry angle determination, the gantry was positioned at 10° intervals from −180° to +180° using the linac readout system. At each gantry angle, while the gantry was static, the gantry was measured using the Double Dot method, OBI gantry angle encoder and digital inclinometer (NG360).

### 2.E Dynamic gantry angle QA plans

**Figure 4:**
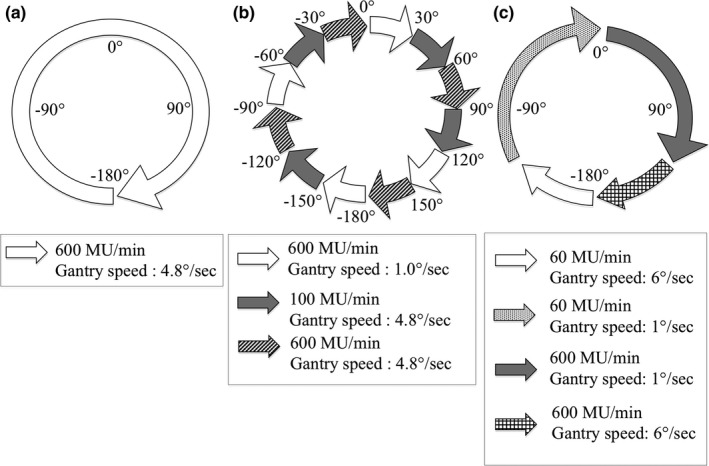
Design of gantry angle QA test plans: constant gantry speed (left), gantry speed transitions (middle), and maximum gantry speed inertia (right).

Three VMAT test plans were designed for gantry angle QA; constant gantry speed, gantry speed transitions, and maximum gantry speed tests (see Fig. 4). These plans were created using MATLAB to manually edit a set of VMAT control points. All plans were delivered in clinical mode with both clockwise and counter‐clockwise gantry rotation.

The first test plan, [Fig 4(a)] aims to test the gantry collimation performance with the lowest level of complexity, with constant dose rate (600 MU/min) and gantry speed (~4.8°/s). The second test plan simulates simultaneous changes in dose rate and gantry speed every 30° of gantry rotation [Fig. 4(b)]. The third test plan [Fig. 4(c)] aims to verify the maximum changes in inertia of gantry angle and maximum changes in dose rate with short transition periods. This plan was adapted from the Maximum Allowable Inertia Overshoot (MAIO) test, which is recommended by the Netherlands Commission on Radiation Dosimetry (NCS Report 24, page 26).^8^

During delivery of these plans, gantry angle versus time data was acquired using DynaLog files, the OBI gantry angle encoder and, the Double Dot method. Note that, in dynamic‐gantry mode, inclinometer measurements were not performed due to (a) the low sample rate of the inclinometer (1 Hz) and (b) the interference between the wire connection from the inclinometer and the video.

## 3 RESULTS

### 3.A Accuracy of method for static gantry

**Figure 5:**
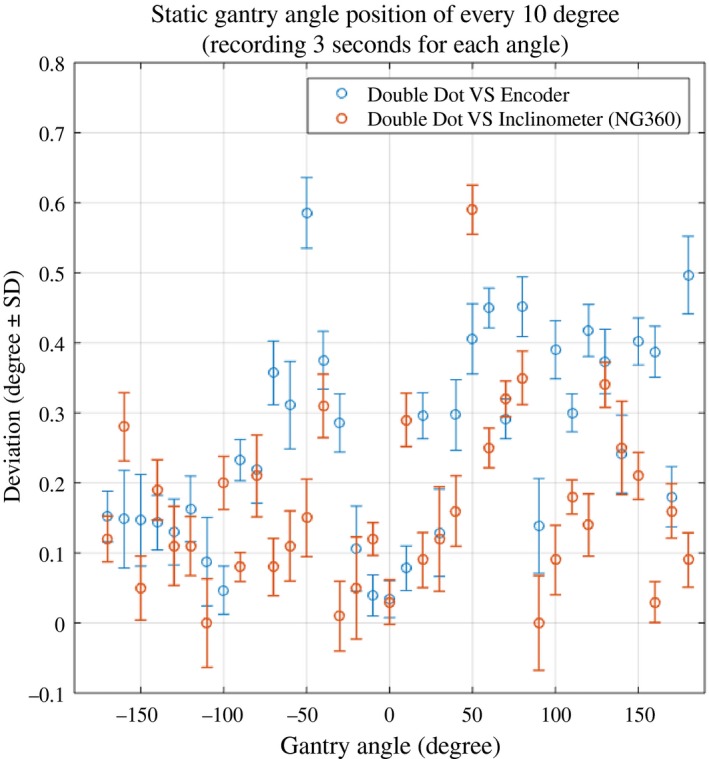
Comparison between Double Dot, encoder and inclinometer gantry angle for static gantry measurements.

Figure 5 shows the absolute deviation between gantry angle determined by our proposed system (Double Dot) against the inclinometer (NG360) and the OBI gantry angle encoder. The average absolute deviation (±1 SD) of the Double Dot gantry angle compared to inclinometer and OBI encoder were 0.16° ± 0.04° and 0.26° ± 0.04°, respectively. The largest observed deviation between the Double Dot method and encoder/inclinometer was approximately 0.6°. Note that, for each individual gantry angle accuracy test, the system used 3 s of video or 90 image frames (30 frames per second) and the gantry angle determination was performed on each frame. The errors bars in Fig. 5 represent the standard deviation of the angles from each of these 90 image frames. The standard deviation was approximately 0.04° for each measurement indicating high stability in the Double Dot measurements.

### 3.B Accuracy during dynamic gantry angle QA plans

**Figure 6:**
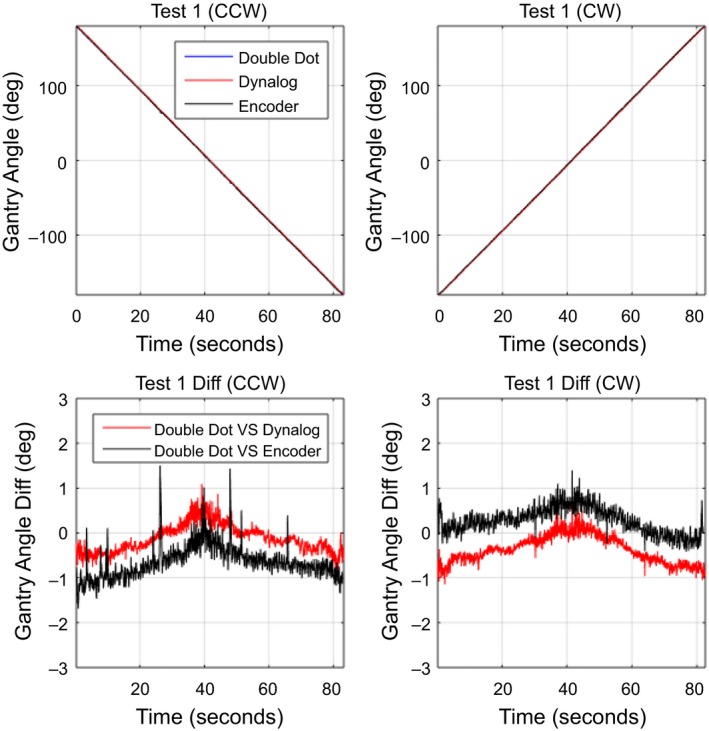
Measured gantry angle results in test plan 1: Constant gantry speed.

**Figure 7:**
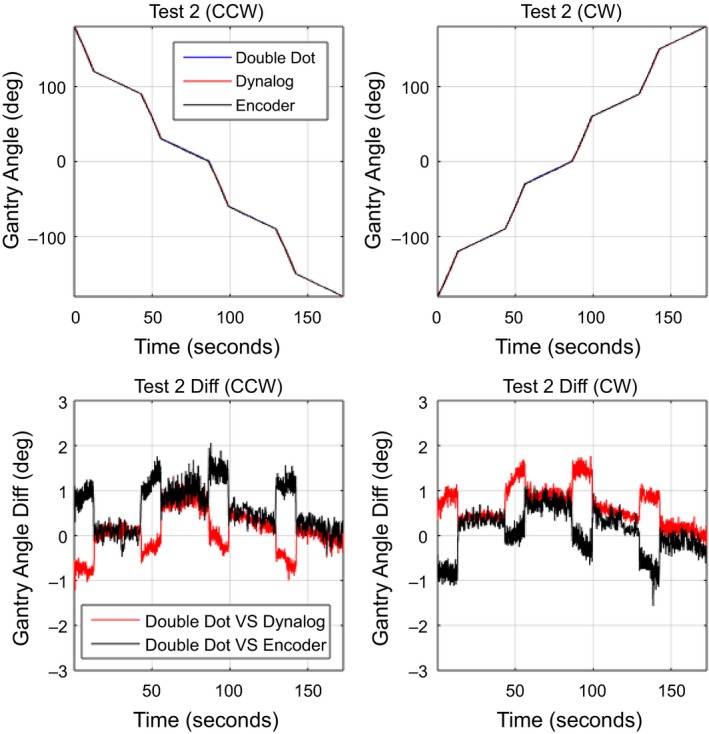
Measured gantry angle results in test plan 2: Gantry speed transitions.

**Figure 8:**
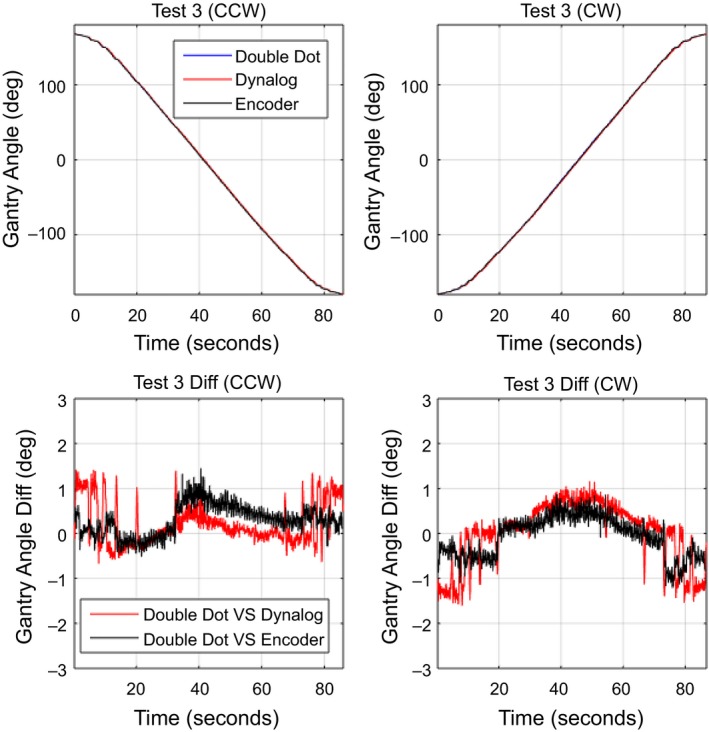
Measured gantry angle results in test plan 3: Maximum gantry speed inertia.

For dynamic gantry deliveries, the Double Dot method was compared to the gantry angle from the DynaLog files and OBI gantry angle encoder for the three QA tests plans. The mean absolute gantry angle difference between the Double Dot Method, DynaLog and encoder is given for each arc in Table 1. The standard deviation of these differences is also shown. The overall accuracy of the Double Dot system compared to DynaLog and encoder in dynamic mode was found to be 0.44° ± 0.32° and 0.47° ± 0.30°, respectively. For the direction of gantry rotation, the average differences and standard deviation were 0.45° ± 0.31° for CCW direction and 0.46° ± 0.31° for CW direction. The maximum absolute gantry angle difference between Double Dot and encoder/DynaLog was 2.1° during test 2. Figures 6, 7, 8 demonstrate the gantry angle comparison of all three test plans.

**Table 1:** Measured gantry angle comparison of Double Dot (DD) to DynaLog and Encoder both counter‐clockwise (CCW) and clockwise (CW) direction

		CCW		CW	
Absolute deviation (°)		Average	1 SD	Average	1 SD
Test1: Constant gantry speed	DD‐DynaLog	0.27	0.18	0.41	0.26
	DD‐Encoder	0.68	0.30	0.32	0.23
Test2: Gantry speed transition	DD‐DynaLog	0.37	0.30	0.67	0.40
	DD‐Encoder	0.66	0.46	0.40	0.28
Test3: Maximum gantry speed inertia	DD‐DynaLog	0.36	0.36	0.55	0.42
	DD‐Encoder	0.37	0.26	0.38	0.25
Average		0.45	0.31	0.46	0.31

## 4 DISCUSSION

A comprehensive time‐resolved VMAT QA using EPID is still associated with some critical issues, one of which is an independent gantry angle check. In our previous studies,^17^, ^19^, ^21^ we have obtained gantry angle information directly from the encoder, which was a limitation of the time‐resolved VMAT QA not being a fully independent of the linac control systems under investigation. Commercial inclinometers are an alternative solution, but current systems are limited in resolution to 1 Hz, which is insufficient for EPID‐based VMAT QA. Currently, available inclinometers also have a lag effect evident at acceleration/deceleration of the gantry.^11^ In this paper, we proposed a fully independent gantry angle measurement using a mobile phone or other small camera with video functionality and double dot pattern printed on a piece of A4 paper. This allows measurement of the time‐resolved gantry angle while dose delivery is being recorded with EPID.

The proposed method is practical and the associated cost is very low as it only requires a small camera (phone) and paper. This method is user friendly, easy to setup, and has high temporal resolution equal to the frame rate of the camera. Here, 30 frames per second were used, but the method can also utilize any higher frame rate resulting in a correspondingly higher temporal resolution. With the acquisition rate, the system can easily be synchronized to EPID images using a linear interpolation method. A limitation of synchronization between EPID and the proposed system, however, still exist in identifying the first frame of beam‐on status. In our system, we assumed that the first frame of gantry rotation represents beam‐on. The accuracy in synchronization between the EPID images and the Double Dot system can be improved by, for example, placing an additional dose rate meter, such as Automess Dose Rate Meter—6150 AD5/6 (Automess, Ladenburg, Germany) next to the mobile phone. Once the radiation is on, the dose rate meter will alarm while video is recoding. This alarm sound can be processed to identify which frame represent “beam‐on” status for synchronization.

The ability to accurately synchronize the video signal with the Dynalog or Encoder signal is difficult. Figure 7 shows that in regions of high gantry speed, a synchronization difference results in larger gantry angle differences between the Double Dot and both Dynalog and Encoder gantry angles. It is also apparent that the encoder always leads the Double Dot and the Dynalog always lags. This produces apparent gantry angle errors in opposite directions and also causes the direction of the gantry angle error to change when direction of gantry angle rotation is reversed as shown in Fig. 7. The impact of this synchronization is also seen in Fig. 6 were a gantry angle difference of 1° is observed at gantry 0° for the dynamic test cases, however, no such error is found for the static gantry measurements in Fig. 5.

In this study, the double dot with red and green colours are used, however, different patterns can also be used. We tested on a green arrow pattern and developed an algorithm to determine the gantry angle based on Hough transform algorithm.^26^ The key is in the image processing method to determine the gantry angle from video. However, we found that the double dot pattern provides highly accurate gantry angle information and is based on a straightforward algorithm, which facilitates an application and implementation. Our proposed double dot pattern has the potential to be permanently attached on the machine. Note that, the size of the dots was also different so that the dot radius could be used as a characteristic to differentiate between the two dots in the images, if colour imaging was not feasible. Moreover, it should be noted that a larger spacing between the dots could improve the gantry angle resolution of the methodology and reduce the noise in the measurement. In this work, we have not investigated the benefit of increasing this spacing as it is convenient and efficient for the two dots to be printed on a standard A4 size piece of paper.

The robustness of image processing techniques in this method is dependent on the camera position. While the exact camera position is not critical, the camera should be roughly aligned with the center of the two dots. Small deviation in this alignment did not result in significant errors. The uncertainty of the video camera signal could also influence the accuracy of gantry angle determination from the system. Higher camera resolution or reducing the framerate could reduce the noise. However, lower framerate will introduce the issue of image blurring from dynamic tests. The systems used for video recording in this study were found to offer adequate image quality for these tests.

In Figs. 6 and 7, the Double Dot showed that the deviation of DynaLog file and Encoder are in opposite direction between CW and CCW during the fast gantry motion (4.8°/s and 5°/s). This pattern may cause from the different timestamps of each method; the Double Dot recorded every 33.3 ms, the DynaLog file recorded every 50.0 ms, and the Encoder recorded every 100 ms. However, the range of variations of all three plans is mostly within the tolerance limit for gantry angle (±1°), which was recommended by Rowshanfarzad et al.*,* (2012). The maximum of deviation between Double dot and Encoder was 2.1° which occurred during Test 2. It is likely that this difference was due to the low temporal resolution of Encoder, which is read out at 10 samples per second, while Double Dot has a higher temporal resolution of 30 samples per second.

It should also be noted that our method is not limited to using a mobile camera, but any other cameras (e.g., a video camera or web camera) can be used. In this study, we proposed the mobile camera for its practicality; (a) it is easily accessible, (b) it can record high‐resolution video, (c) the output video file can be easily transferred to a computer. However, it is not recommended to put the mobile camera close to the radiation beam. When using Double Dot, the room light should be turned on to increase the image contrast and to reduce image noise.

## 5 CONCLUSION

A low cost and independent gantry angle measurement tool using a mobile camera and double dot pattern was developed and its accuracy was evaluated in this paper. The advantage of our method is that allows a simultaneous independent measurement of EPID dosimetry, geometry and gantry angle in a single delivery. The Double Dot pattern is placed on the linac, and its motion due to gantry rotation was recorded using a mobile camera on the treatment couch. The gantry angle can be automatically measured offline from the video file using image processing techniques. The accuracy of this method in static mode was 0.26° ± 0.04° on average compared to inclinometer and encoder. The proposed three test plans were used to evaluate the system performance for dynamic gantry rotation, and an accuracy of 0.46° ± 0.31° was found when comparing to DynaLog file and encoder. Our proposed gantry angle measurement tool is an accurate and practical solution for VMAT QA as it provides a fully independent gantry angle measure while still allowing dosimetric testing using EPID, using low‐cost equipment, and an easy setup.

## CONFLICT OF INTEREST

The authors declare no conflict of interest.
